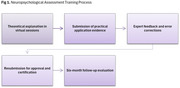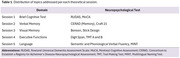# Implementing a Training Program for Cognitive and Neuropsychological Assessments in a Latin America country

**DOI:** 10.1002/alz70858_105516

**Published:** 2025-12-26

**Authors:** Graciet Verastegui, Diego Bustamante‐Paytan, Rosa Montesinos, Nilton Custodio, Giuseppe Tosto

**Affiliations:** ^1^ Unidad de Investigación de Deterioro Cognitivo y Prevención de Demencia, Instituto Peruano de Neurociencias, Lima, Peru, Lima, Lima, Peru; ^2^ Unidad de Investigación de Deterioro Cognitivo y Prevención de Demencia, Instituto Peruano de Neurociencias, Lima, Lima, Peru; ^3^ Universidad de San Martín de Porres, Facultad de Medicina, Centro de Investigación del Envejecimiento, Lima, Lima, Peru; ^4^ Unidad de Investigación y Docencia, Equilibria, Lima, Peru; ^5^ Taub Institute for Research on Alzheimer's Disease and the Aging Brain, Vagelos College of Physicians and Surgeons, Columbia University, New York, NY, USA; ^6^ Gertrude H. Sergievsky Center, Taub Institute for Research on the Aging Brain, Departments of Neurology, Psychiatry, and Epidemiology, College of Physicians and Surgeons, Columbia University, New York, NY, USA; ^7^ Department of Neurology, College of Physicians and Surgeons, Columbia University, and the New York Presbyterian Hospital, New York, NY, USA; ^8^ G.H. Sergievsky Center, Vagelos College of Physicians and Surgeons, Columbia University, New York, NY, USA

## Abstract

In the coming decades, the number of dementia cases is projected to increase worldwide. This public health issue will significantly impact low‐ and middle‐income countries, such as those in Latin America, due to their aging populations. Proper diagnosis and identification of these patients require a comprehensive assessment, including neuropsychiatric, laboratory, and imaging evaluations, supported by neuropsychological assessments.

However, despite the necessity of neuropsychological evaluations to identify impairments in one or more higher cognitive functions—facilitating the differentiation between various types of dementia—the availability of trained personnel to administer these assessments correctly is limited. This shortage leads to undiagnosed cases or inaccurate results in clinical studies. Here, we describe and propose a structured virtual training model for future raters assessing dementia patients, based on the training conducted at the Instituto Peruano de Neurociencias as part of the GLASS‐AD (Global Latinos Sequencing Study for Alzheimer's Disease) study (Grant number: U01‐AG081817).

The primary objective of this training was to ensure that raters could accurately administer neuropsychological tests, thereby guaranteeing the validity of results. The training was structured into several phases: (1) Virtual sessions provided a theoretical explanation of neuropsychological tests, organized by cognitive domains. (2) Participants submitted evidence of their practical application of the material, using cognitively healthy individuals simulating cognitive impairment symptoms. (3) Expert neuropsychologists reviewed the trainees' submitted videos, providing feedback and corrections. (4) Participants then resubmitted a video with the corrected test administration for approval and certification. (5) Finally, a follow‐up evaluation was scheduled six months later, requiring the submission of a new video to verify the quality and consistency of test administration.

Standardizing training is essential to ensure that personnel are adequately prepared, improving the identification and diagnosis of dementia patients. This facilitates timely treatment and the development of strategies to mitigate the disease's impact on families and communities while also helping reduce the costs associated with patient care, especially in LMICs.